# Addenbrooke's Hospital

**Published:** 1902-02-15

**Authors:** 


### ADDENBROOKE'S HOSPITAL.

In June, 1898, Sir Henry Burdett made a report on the condition of Addenbrooke's Hospital (*vide* The Hospital, page 276, July 16th, 1898, and page 314, February 10th, 1900), in which was embodied the proposals for administrative alterations referred to below. His advice was not adopted at the time, and other schemes have since been tried which have not proved satisfactory. The present acceptation of his suggestions shows that Sir Henry Burdett was slightly in advance of public opinion in Cambridge three years ago.

RECOMMENDATIONS CONCERNING THE GOVERNMENT OF THE ADDENBROOKE'S HOSPITAL, CAMBRIDGE.

The Advisory Council have had under consideration the question of a change in the scheme of government of the hospital.

An opinion exists among the governors that the management of the hospital should be vested in a more permanent and less fluctuating body than the existing weekly board. Such a course was suggested in the Report of the special committee on Sir Henry Burdett's Report, dated September 30th, 1898, and their recommendation that a committee of management consisting of 18 governors, together with a chairman, should be appointed annually, whose duties should include those then assigned to the weekly board, the select governors, and the finance committee, was carried on a poll, taken on November 28th, 1898, which resulted in 145 votes for the motion, and 95 against. A legal opinion was subsequently obtained to the effect that the appointment of such a committee by the governors was not within the powers conferred by the Act of Parliament Geo. III. (1761).

The council share in principle the opinion then expressed by the majority of the governors. They think it desirable that the management of the hospital should be vested in a committee appointed by the governors, such committee to hold weekly meetings for the despatch of ordinary routine business, for which a certain quorum should be necessary, and to hold monthly meetings for the administration of the general concerns of the hospital, for which a larger quorum should be required.

No important alteration seems to be called for in the constitution, as defined in the rules, orders, and bye-laws of the hospital, numbered 1 to 8 inclusive, which describe the government of the hospital as placed in the existing corporate body and which give a definition of the term governor.

It appears advisable to retain the practice of holding at least four general courts of the governors in every year. A discretion as to the place and time of meeting is, however, to be desired. Occasions may arise when the board room at the hospital will be too small, and the dates and times of meeting of the existing quarterly courts are not convenient for the University governors.

The representative character of the existing advisory council has given an element of strength to that body; and it would appear desirable to retain that character if a general administrative committee is appointed to take the place of the present advisory council. Such a committee might consist of 18 members. The period for which it should be appointed, whether for one year or for several, will have to be considered. It would give more continuity to the management, if members were elected for three years, one-third of the number going out of office every year.

The existing system, by which any rule or bye-law can be suspended or altered, without notice, at any general court, places the good government of the hospital on a precarious footing. Changes may, however, from time to time be required, some of a fundamental, others of an administrative, character. These might all be dealt with by the governors in general court, the resolutions by which they are respectively effected being passed with certain formalities, insuring proper notice to the governors and sufficient time for a due consideration of the proposed changes.

With reference to the manner in which the changes here suggested can be effected the council are advised that the authority of Parliament is necessary. The Charitable Trusts Act, 1853, provides a method by which an amending scheme may with the co-operation of the Charity Commissioners be obtained without expense to the hospital. The mode of procedure is very similar to that required for obtaining a provisional order under the Public Health Acts, and involves a local inquiry, held by one of the inspectors of the Board of Charity Commissioners, where the nature of the case requires it. The scheme, when finally approved and certified by the Board, is reported to both Houses of Parliament; and if it is enacted by any Act of Parliament that such scheme shall be confirmed and take effect, either with or without any alterations or modifications thereof, every such scheme is to be deemed a public general Act.

The council subjoin some clauses and outlines of clauses in order to give a clearer idea of the changes they would suggest; but it is not necessary for them at this stage to make any other recommendation than that the general principle involved in the clauses be approved, with a view to a complete scheme being prepared to be considered at a future court before submission to the Charity Commissioners.

1. The constitution of the hospital as described in the first eight of the rules, orders and bye-laws of the hospital is to remain unaltered, except that the words, "and weekly boards," in clause 6 shall be struck out.

2. The governors in general court may from time to time alter the day and the month, on which any of the four general courts is appointed by the Act to be held, and they may also change the hour and place of meeting. The times for holding the said courts shall be fixed annually at the first general court to be held in the last three months of the civil year.

3. A special general court shall be called whenever it is required by resolution of the general committee hereinafter mentioned.

4. A general committee shall be established consisting of 18 elected governors: 6 from the County, 6 from the Borough, and 6 from the University. The election to be conducted on lines similar to those prescribed with respect to the advisory council.

5. A chairman and vice-chairman of the general committee shall be annually appointed by the general committee.

6. Subject to the provisions of this scheme, and to the bye-laws, being not inconsistent therewith, the administration of the hospital shall be conducted and carried on, and all its affairs shall be managed, by the general committee. Provided always that the following acts and proceedings shall always be subject to confirmation by a general court, that is to say, the purchase or sale of any securities of the hospital; the incurring of any liability of an extraordinary character exceeding at any one time the sum of £50; the election of members of the medical, or surgical, or dental staff, or of the treasurer, secretary, superintendent, or matron. The general committee shall bring up a report of its proceedings at each of the four statutory general courts.

7. The general committee may at any time suspend any officer from the duties of his or her office.

8. The general committee shall meet at the hospital once in each week for the despatch of business. Five members shall form a quorum on the first weekly meeting in each month, and at any specially summoned meeting; at all other meetings three shall be the quorum.

9. The general committee shall have power to make rules for the regulation of its own proceedings, and those of any special committee appointed by it.

10. No motion out of the usual routine, which may affect the finances of the hospital, or its rules and customs, shall be decided at the ordinary weekly meeting.

11. Any member present at an ordinary weekly meeting of the general committee may require that a matter brought forward, which in his opinion should be deferred to the consideration of an extraordinary meeting or of a monthly meeting, shall be so deferred. And if one other member present concur, such course shall be adopted.

12. Special committees may be appointed by any general court, or by the general committee, for the purpose of considering and reporting upon any subject connected with the hospital; and any person willing to act, whether connected with the hospital or not, may be appointed a member of a special committee.

13. Alterations of this scheme may be made by special resolution as hereinafter defined, such resolution being confirmed by a majority at a special court to be held not less than 14, nor more than 28, days after the passing thereof.

14. Alteration of rules, orders or bye-laws may be made by special resolution. A resolution shall be deemed special, which is passed by a majority of not less than two-thirds of the governors present at a general court, not less than 20 days' notice of the intention to propose such resolution having been given.

15. All provisions of the Act of George III. (1761) which are inconsistent with this scheme shall be repealed.

The financial condition of the hospital was also referred to the advisory council for their consideration. The council think that some addition might properly be made to the income of the hospital by charging out-patients with the cost, or part of the cost, of their medicines; and by making special provision for the reception within the hospital of patients to whom a charge can be made for their treatment. In any scheme which may be submitted to the charity commissioners it should be made clear that the governors are not precluded from passing bye-laws for these purposes.

